# Functional Analyses in Patient‐Derived Neurons Establish Pathogenicity for *STXBP1* Splice Variant c.429+5G>A

**DOI:** 10.1155/humu/1448702

**Published:** 2026-06-16

**Authors:** Sylvia Korhorn, Additya Sharma, Jan J. Sprengers, Shilpa Anand, Jennifer R. Ramautar, Klaus Linkenkaer-Hansen, Hilgo Bruining, Ruud F. Toonen, Matthijs Verhage, Mala Misra-Isrie

**Affiliations:** ^1^ Department of Functional Genomics, Center for Neurogenomics and Cognitive Research (CNCR), Vrije Universiteit Amsterdam, Amsterdam, the Netherlands, vu.nl; ^2^ Department of Integrative Neurophysiology, Center for Neurogenomics and Cognitive Research (CNCR), Vrije Universiteit Amsterdam, Amsterdam, the Netherlands, vu.nl; ^3^ N=You Neurodevelopmental Precision Center, Amsterdam Neuroscience, Amsterdam Reproduction and Development, Amsterdam UMC, Amsterdam, the Netherlands, amc.nl; ^4^ Child and Adolescent Psychiatry and Psychosocial Care, Emma Children′s Hospital, Amsterdam UMC, Amsterdam, the Netherlands, amc.nl; ^5^ Department of Human Genetics, Amsterdam UMC, Amsterdam, the Netherlands, amc.nl

**Keywords:** case report, functional validation, IPSC-derived neurons, spice-site variant, STXBP1-RD

## Abstract

Pathogenic *STXBP1* variants cause a broad spectrum of neurodevelopmental disorders. We investigated a patient with developmental delay but no seizures, carrying a heterozygous, predicted splice site variant, c.429+5G>A, initially classified as a variant of uncertain significance. Patient‐derived neurons had normal morphology in vitro, but > 40% reduced MUNC18‐1/*STXBP1* protein and mRNA levels, comparable with two established loss‐of‐function variants (Asp262Val and Arg235*). Nonsense‐mediated decay inhibition increased transcript levels, and RT‐PCR/minigene analysis demonstrated Exon 6 skipping, resulting in a frameshift and premature stop codon. Relative to a large cohort of typically developing children, EEG biomarker analysis revealed elevated long‐range temporal correlations in beta and gamma bands, increased delta power, and reduced excitation/inhibition ratio in the beta band. This multimodal assessment demonstrates that c.429+5G>A is a disease‐causing variant, and the value of combining functional and clinical data for accurate variant interpretation. Based on this, the patient was included in the EU *STXBP1* registry ESCO.

## 1. Introduction

Pathogenic de novo variants in *STXBP1* cause a broad spectrum of neurodevelopmental disorders, collectively termed STXBP1‐related disorders (STXBP1‐RD) [1, 2]. *STXBP1* encodes the protein MUNC18‐1, a key regulator of synaptic vesicle fusion by orchestrating SNARE complex assembly. As such, MUNC18‐1 is indispensable for neurotransmitter release and neuronal communication [3, 4].

Clinically, STXBP1‐RD is characterized by marked phenotypic heterogeneity. Although early‐onset epilepsy is a prominent feature in most affected individuals, seizure‐free patients have also been reported, and no clear genotype–phenotype correlations have been established to explain the presence or severity of seizures [2, 5]. Accumulating evidence indicates that haploinsufficiency is the primary disease mechanism [6–10]. The mutational spectrum is diverse, with approximately 48% of pathogenic variants classified as missense, 22% as protein truncating, and 15% predicted to affect splicing [2]. Splice‐altering variants may disrupt canonical splice sites, resulting in aberrant mRNA processing and, in many cases, mRNA degradation by nonsense‐mediated decay (NMD) [11].

Here, we describe a female patient carrying an uncharacterized heterozygous *STXBP1* splice variant, c.429+5G>A. In silico analysis using Human Splice Finder predicts loss of the Exon 6 donor splice site. Using patient‐derived induced neurons (iNeurons) and a panel of functional assays, we demonstrate that this variant indeed disrupts RNA splicing and leads to reduced MUNC18‐1 protein levels. By benchmarking the patient′s cellular phenotype to two healthy controls and two previously characterized patient variant lines [9], we provide further support for this mutation. Although splice variants in *STXBP1*, including variants affecting the same splice site [12], have been reported previously [1, 2], this study provides the first comprehensive characterization of a *STXBP1* splice variant with both functional and clinical evidence, bridging the gap between genomic prediction, cellular pathology, and clinical phenotypes.

## 2. Results

### 2.1. Clinical Presentation

The patient was born at term with birthweight of 2770 g (p3) and Apgar scores of 7 and 9. A caesarean section was performed because of breech presentation. She had co‐occurring milk allergy with reflux and severe eczema. Early developmental milestones were delayed, with independent ambulation and the production of first words both occurring at approximately 2 years of age. There was no history of seizures, and family history was unremarkable. She had hypermobility, but no obvious dysmorphic features. At the age of 4, she was referred for genetic testing to analyze possible causes for the developmental delay.

**Figure 1:**
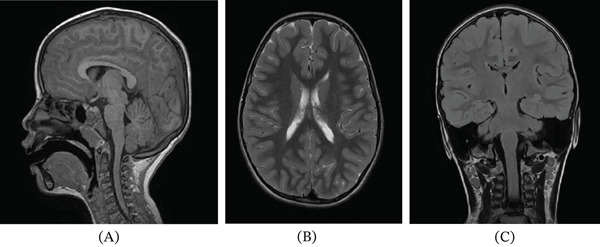
Brain MRI of index case. (A) Sagittal T1‐weighted, (B) axial T2‐weighted, and (C) coronal T2‐weighted images obtained at age 5. Brain structures appeared normal with no clear abnormalities.

SNP‐array and Fragile‐X screening showed no abnormalities. Magnetic resonance imaging revealed no structural brain abnormalities (Figure 1A–C). However, trio‐based exome sequencing revealed a heterozygous de novo variant in the *STXBP1* gene (c.429+5G>A). The variant was in the donor splice site of Exon 6 with possible deleterious effect on splicing. However, it was located outside the canonical ± 1–2 splice site positions without proven functional effect. The variant was absent from the Genome Aggregation Database (gnomAD). The variant had not been described before in patients with STXBP1‐RD and was reported once before in ClinVar as a variant of uncertain significance. Since it was also not a perfect match with the traditional clinical phenotype of STXBP1‐RD due to absence of seizures, it was classified as a variant of uncertain significance. To clarify this, the case was subjected to the N = You standard multimodal assessment pipeline, including blood sampling, PBMC isolation followed by iPSC generation, mRNA and protein analyses in synaptically mature iPSC‐derived neurons, clinical assessments, cognition, adaptive behavior, and resting‐state EEG.

### 2.2. MUNC18‐1 Protein Levels Are Reduced in c.429+5G>A Patient iNeurons

**Figure 2:**
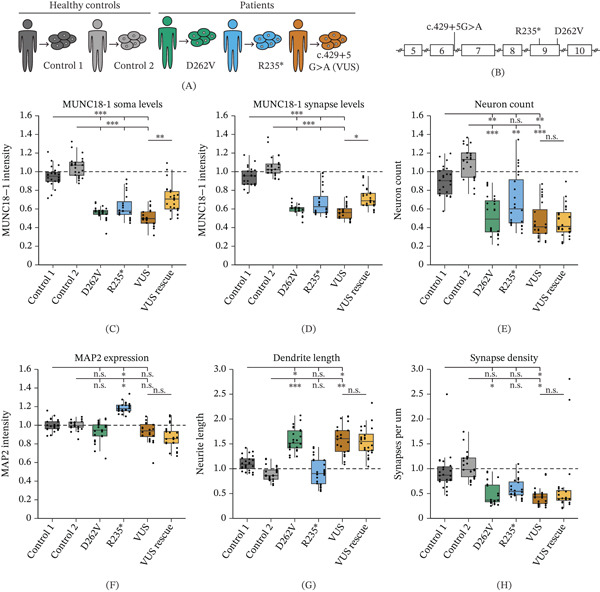
Reduced protein levels found in patient‐derived cell lines, including the VUS line. (A) A schematic of the cell lines used and (B) their mutation locations in *STXBP1*. Decreased protein levels of all patient lines were observed in (C) cell soma and (D) synapses compared with both control lines. (E) A decreased number of neurons was measured using DAPI staining and MAP2 positivity. (F) MAP2 expression was in generally the same in all conditions, with only a slight increase in R235∗ iNeurons. (G) The average dendritic length was increased in D262V and VUS lines compared with controls, with no difference measured in R235∗. (H) Decreased synaptic density was found in the D262V and VUS cell lines, with no difference measured in R235∗.

To assess the functional impact of the c.429+5G>A variant, iPSC‐derived neurons were generated from the index patient and analyzed alongside two healthy control lines and two previously characterized STXBP1‐RD patient lines Asp262Val (D262V) and Arg235∗ (R235∗) (Figure 2A,B).

**Figure 3:**
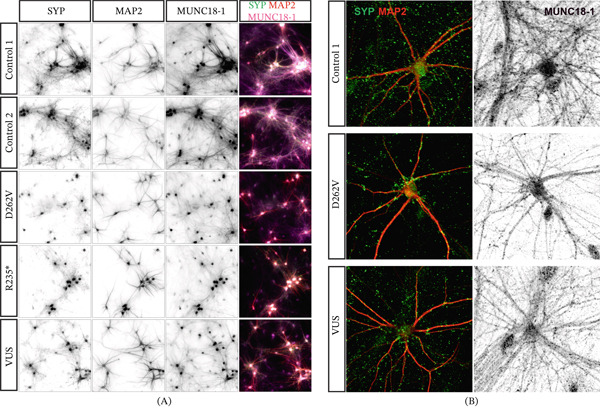
Representative images of human neurons carrying *STXBP1* variants. Shown are (A) widefield images of network cultures and (B) higher magnification confocal images of single neurons.

Immunocytochemical analysis using antibodies against the dendritic marker MAP2, the synaptic vesicle marker synaptophysin‐1 (SYP), and MUNC18‐1 showed a marked reduction in MUNC18‐1 protein levels in neurons derived from the index, comparable with the decreases observed in the D262V and R235∗ patient lines (Figures 2C,D and 3A,B). Although the number of MAP2‐positive neurons was higher in both control lines versus the patient‐derived lines (Figure 2E), MAP2 signal intensity did not significantly differ between groups, only R235∗ (Figure 2F). Analysis of dendritic morphology showed that neurons from both the D262V and index case displayed increased dendritic length compared with controls, whereas the R235∗ neurons were more similar to control lines (Figure 2G). Furthermore, synaptic density, quantified as the number of SYP‐positive puncta per micrometer of neurite length, was reduced in patient‐derived neurons relative to wild‐type controls (Figure 2H) for D262V and the index case. Taken together, these findings demonstrate that all patient‐derived neurons exhibit reduced MUNC18‐1 expression and plating efficiency, accompanied by lower neuronal density and increased dendritic length in the case of D262V and the index case.

### 2.3. Aberrant mRNA Processing Caused by the VUS Allele Leads to NMD

**Figure 4:**
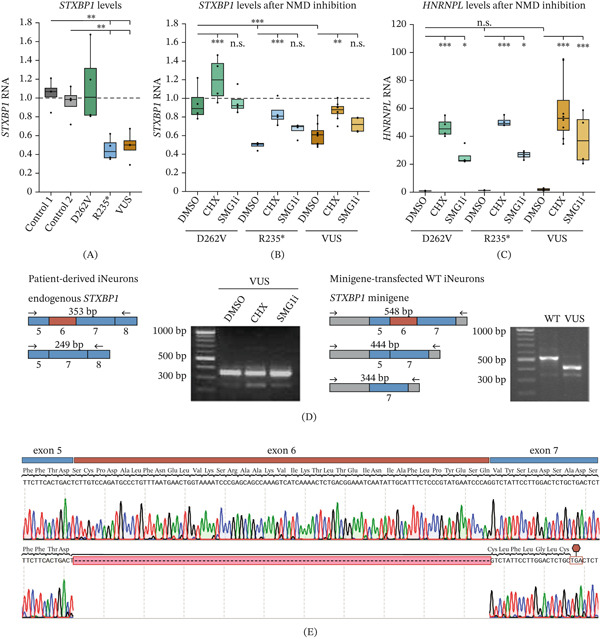
VUS variant causes aberrant splicing and mRNA degradation. (A) Decreased mRNA levels were observed in R235∗ and VUS cell lines compared with controls, whereas the D262V line showed levels comparable with controls. (B) Inhibition of NMD with CHX and SMG1i resulted in increased RNA levels in R235∗ and VUS lines, whereas only CHX increased RNA levels with the D262V line. (C) RNA levels of the NMD positive control *HNRNPL* were greatly increased upon CHX and SMG1i treatment. (D) RT‐PCR using primers spanning Exons 5–8 revealed two PCR products in VUS patient iNeurons. Consistently, the minigene harboring the VUS variant produced a shorter splice product compared with the WT minigene. (E) Sequencing results from both patient‐derived and minigene transfected iNeurons confirmed exclusion of Exon 6, resulting in a frameshift and the introduction of a premature termination codon.

The reduced MUNC18‐1 protein levels observed in the index case neurons, together with the intronic location of the variant, suggested altered mRNA processing rather than a direct coding effect. Quantification of *STXBP1* mRNA levels revealed a significant reduction in RNA expression in iNeurons derived from the VUS and R235∗ patients compared with healthy controls. As expected, RNA levels were not affected by the missense D262V line (Figure 4A). These findings suggest that transcripts harboring the VUS undergo transcriptional or post‐transcriptional alterations, resulting in decreased mRNA levels.

To test the involvement of NMD, patient‐derived neurons were treated with the NMD inhibitors cycloheximide (CHX) and SMG1i (SMG1i inhibitor [13]). NMD inhibition with CHX led to increased *STXBP1* mRNA levels in all cell lines, whereas SMG1i treatment elevated *STXBP1* levels only in the VUS and R235∗ lines, indicating that NMD contributes to the loss of these transcripts (Figure 4B). Notably, CHX may have additional effects beyond NMD inhibition. *HNRNPL* expression served as a positive control for NMD inhibition and showed a marked (50 and 25 fold) increase upon CHX and SMG1i treatment (Figure 4C).

To investigate the mechanism further, RT‐PCR analysis following NMD inhibition revealed two distinct PCR products in the index case‐derived neurons (Figure 4D). Consistent with this finding, a minigene construct encompassing *STXBP1* and their intervening introns produced a shorter splice product when the VUS was introduced and transfected into wild‐type neurons. Sequencing of PCR products from both patient‐derived neurons and minigene‐transfected cells confirmed skipping of Exon 6, resulting in a frameshift and the introduction of a premature termination codon (Figure 4E). Taken together, these results demonstrate that the VUS disrupts splicing at the donor site of Exon 6, leading to exon skipping, a frameshift with premature termination, and degradation of the transcript via NMD.

### 2.4. Developmental Assessments

Because these cellular assays provided strong evidence to conclude this variant is pathogenic, the patient was assessed again, now including adaptive behavior and IQ analyses. At the age of 8 years, the patient produces simple sentences but continues to experience difficulties with concentration and sensory processing. She attends a school for special education. She has been diagnosed with attention deficit hyperactivity disorder and exhibits several features consistent with autism spectrum disorder. She is not yet toilet trained. Because of sleep disturbances, she receives treatment with melatonin.

Her IQ scores were between 52 and 68 (SON‐R 2‐8 FIQ, performance score 52–71, reasoning score 54–77), congruent with her adaptive development (Vineland‐3 Adaptive Behavior Composite 60–66). Treatment with methylphenidate resulted in increased irritability and anger outbursts and was stopped after a month.

**Figure 5:**
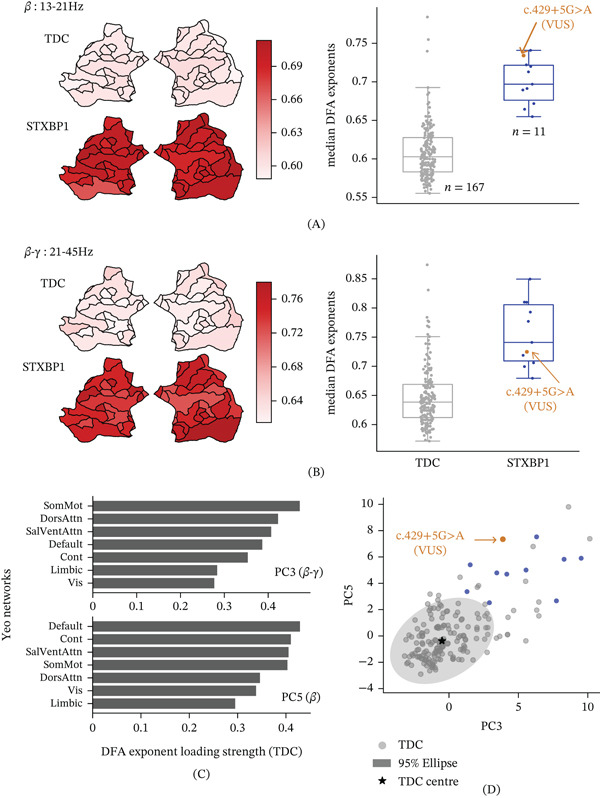
Brain activity patterns of the index case resemble the STXBP1 patient group. (A) Cortical maps showing distribution of EEG‐derived detrended fluctuation analysis (DFA) exponents in beta band (*β*: 13–21 Hz) for typically developing children (TDC) and STXBP1 patients across brain regions. (B) Cortical maps showing distribution of EEG‐derived DFA exponents in upper beta–gamma band (*β* − *γ*: 21–45 Hz) for TDC and STXBP1 patients across brain regions. (C) DFA exponent feature loading distribution across Principal Component 3 (PC3) and Principal Component 5 (PC5) for TDC. Each feature represents the loading strength of DFA exponent for *β*‐*γ* (PC3) and *β* (PC5) across cortical Yeo networks (DorsAttn: dorsal attention; Vis: Visual; Cont: Control; SomMot: somatomotor; SalVentAttn: salience ventral attention). (D) Projection space of TDC DFA exponent PC3 and PC5 components. Gray points indicate position of individual TDC. Black cross indicates robust center of the projection space based on TDC. The index patient (c.429+5G>A) is denoted by an orange color dot.

**Figure 6:**
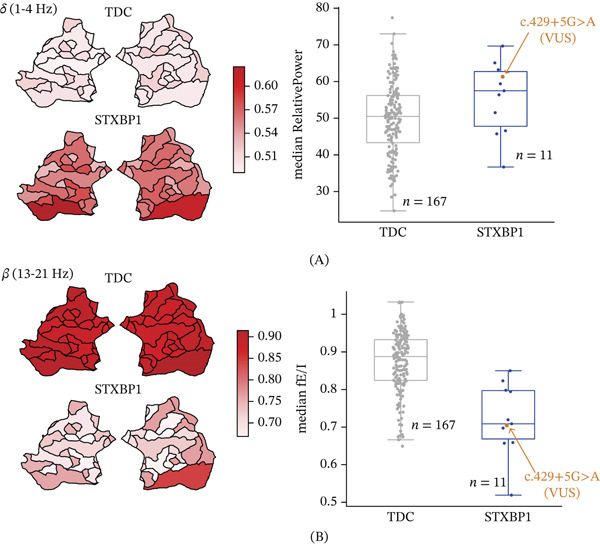
Reduced excitation–inhibition balance in index case resembles the STXBP1 patient group. (A) Cortical maps showing distribution of EEG‐derived low‐frequency relative delta (1–4 Hz) power for typically developing children (TDC) and STXBP1 patients across brain regions. (B) Cortical maps showing distribution of EEG‐derived beta band (13–21 Hz) functional excitation–inhibition (fE/I) ratio for TDC and STXBP1 patients across brain regions. The index patient (c.429+5G>A) is denoted by an orange color dot.

Quantitative analysis of resting‐state EEG was performed by comparing relative power, long‐range temporal correlations indexed with detrended fluctuation analysis (DFA), and functional excitation/inhibition ratio between TDC and an STXBP1‐RD patient cohort [14], including the index case. The index case exhibited elevated long‐range temporal correlations in the 13–45‐Hz frequency range across resting‐state brain networks compared with TDC (Figure 5A,B). Dimensionality reduction based on PCA (see Methods) revealed a clear separation from the normative space of a typically developing cohort along principal components PC3 and PC5 capturing DFA in the upper and lower beta bands, respectively (Figure 5C,D). This finding is in line with our previously investigated STXBP1‐RD patients [14]. Additionally, relative power in the delta band (1–4 Hz) was increased and fE/I ratio in the lower beta band (13–21 Hz) was decreased across brain networks compared with TDC (Figure 6A,B).

## 3. Discussion

In this study, we identified a previously uncharacterized splice variant in *STXBP1* and performed a comprehensive characterization of its cellular consequences using patient‐derived neurons. We demonstrate that this variant leads to aberrant RNA splicing with skipping of Exon 6, resulting in the introduction of a premature termination codon and subsequent degradation of the mutant transcript via NMD. Consistent with this mechanism, patient‐derived iNeurons exhibited reduced *STXBP1* mRNA and MUNC18‐1 protein levels. Together, these findings indicate that the variant acts as a loss‐of‐function allele through transcript degradation and reduced protein expression.

Reduced MUNC18‐1 levels have previously been shown to impair cellular function in patient‐derived and rodent neurons, supporting haploinsufficiency as the disease mechanism in STXBP1‐RD [7–9]. The observed reduction in MUNC18‐1 protein levels in patient‐derived neurons reported here provides direct functional evidence supporting the pathogenicity of this splice variant, shifting its classification from a variant of uncertain significance toward a likely pathogenic variant.

To place the cellular phenotype of this variant in a broader context, we compared the VUS line with two previously characterized STXBP1 patient‐derived iNeurons (D262V and R235∗). All patient‐derived neurons showed decreased neuron counts compared with control lines, despite identical initial plating densities. This reduced plating efficiency observed in patient neurons may reflect differences in culture history rather than a reduced viability of patient neurons. Control lines underwent many more passages, and selection for strong adhesive properties has probably occurred. Moreover, after initial plating differences, no further indications for reduced viability or neuron loss were observed throughout in vitro maturation (up to 6 weeks in vitro).

Dendritic length was significantly increased in both D262V and VUS neurons and synaptic density was reduced in both, but not in R235∗. Given the overall lower neuronal density in patient cultures, the effect in D262V and VUS lines may reflect decreased network complexity rather than a primary defect in synaptogenesis. This interpretation is supported by previous experiments on the R235∗ and D262V lines, where no differences in dendritic length and synapse density where observed [9]. These earlier experiments were performed after a longer culture period, suggesting that some of the phenotypes observed here may represent developmental delays rather than persistent structural deficits. The absence of morphological or synaptic alterations in R235∗ neurons may indicate a milder structural phenotype or a difference in developmental timing compared with the other patient lines.

The EEG analysis identified neurophysiological abnormalities in the index patient consistent with the electrophysiological signature previously described in STXBP1‐RD. Elevated DFA exponents in the 13–45‐Hz range, reflecting a highly complex temporal variation in beta–gamma activity as we have reported previously [14, 15]. Normative projection analysis of the DFA exponents revealed that deviations were more pronounced in the lower beta band (13–21 Hz) than in the upper beta–gamma range (21–45 Hz), suggesting that the abnormal temporal structure of oscillations is not uniformly distributed across this frequency range but preferentially expressed at lower beta frequencies. Decreased fE/I in the lower beta band further supports a shift toward inhibition‐dominated network dynamics, consistent with the inhibition‐dominated resting‐state signature by Houtman et al. [14]. The elevated delta relative power similarly accords with prior qEEG evidence of diffuse slow‐wave excess in STXBP1‐RD, where frontal delta activity has been identified as a disease‐specific feature correlating with phenotypic severity [14–16]. Together, these qEEG analyses provide independent, noninvasive evidence of altered cortical network dynamics in this patient, complementing the cellular evidence of MUNC18‐1 haploinsufficiency and strengthening the overall case for variant pathogenicity.

From a clinical perspective, the identification and functional validation of splice‐altering *STXBP1* variants are highly relevant, given the broad phenotypic spectrum associated with STXBP1‐RD and therefore difficulty in interpreting rare *STXBP1* variants in the absence of a defining phenotype. Our findings underscore the importance of functional assays in patient‐derived neurons for variant interpretation, particularly for splice variants that are difficult to classify based on in silico prediction alone. In addition, the EEG findings provide complementary evidence of altered brain dynamics, strengthening the clinical interpretation of the variant. By providing mechanistic evidence of loss of function at both the RNA and protein level, together with clinically relevant EEG abnormalities and overlapping cognitive scales, this study illustrates how multimodal functional phenotyping can directly impact clinical variant classification and improve diagnostic certainty for affected individuals, family members, caregivers, and other stakeholders. Accurate variant classification is a crucial prerequisite to accurately describe the natural progression of STXBP1‐RD and for the meaningful evaluation of candidate intervention strategies. Given our overall conclusions, the index patient was admitted to the EU *STXBP1* registry ESCO (http://www.stxbp1eu.org).

## 4. Methods

### 4.1. Whole‐Exome Sequencing (WES)

DNA from the index case was enriched using the Agilent SureSelectXT Human All Exon V7 capture kit and paired‐end sequenced on the Illumina platform (outsourced). Sequencing data were demultiplexed with bcl2fastq2 Conversion Software from Illumina. Illumina DRAGEN Bio‐IT Platform was used for read mapping to the hg19 genome and sequence variant detection. Subsequently, variants were filtered with the Alissa Interpret software package (Agilent Technologies) on quality (read depth ≥ 10), frequency in databases (gnomAD [v2.1]) and location (within an exon or first/last 10 bp of introns). Initially, a gene panel of > 1200 genes associated with intellectual disability was applied, followed by trio‐based open exome analysis. Variants were further selected based on three inheritance models (de novo autosomal dominant, autosomal recessive, and X‐linked recessive).

### 4.2. Ethical Guideline Compliance

This study was conducted in accordance with the Helsinki Declaration, in a diagnostic setting. We obtained written informed consent from parents of the patient for publication. Collection of skin biopsies was approved as described previously [9] (METc 2018.329, Amsterdam UMC), and blood sample collection was approved under METc 2022‐13846 (Amsterdam UMC).

Primary astrocytes were prepared from Wistar rat pups (Crl:WI, Strain Code 003), bred according to international and national regulations for animal experiments in the Netherlands.

### 4.3. Generation of Induced Pluripotent Stem Cells (iPSCs)

Control iPSC lines where BIONi010‐C (RRID:CVCL_1E68, EBiSC/Bioneer) (Control 1) and GM23973 from Coriell Institute for medical research (RRID:CVCL_BW42, NJ, USA) (Control 2), both previously characterized [17, 18]. Patient‐derived lines carrying *STXBP1* variants D262V and R235∗ were generated from skin biopsies following the reprogramming protocol described in Van Berkel et al. (2024). Blood samples from the index case were sent to the Radboudumc Stem Cell Technology Center for episomal reprogramming. To confirm genomic integrity, copy number variation (CNV) analysis by WES was performed on DNA isolated from both parental blood and derived iPSC clones. Presence of the mutations in the iPSC lines was confirmed by Sanger sequencing.

### 4.4. CNV Analysis iPSC Lines

For CNV screening, genomic DNA was isolated using the ReliaPrep gDNA Tissue Miniprep System (Promega) and submitted to the Global Screening Array Consortium at Erasmus MC, Rotterdam. DNA was analyzed using the Illumina GSAMD v3 beadchip platform (GRCh37). Clones were excluded if CNVs spanning more than 10 SNPs exceeded 500 kb and had a confidence score > 20. CNVs between 25 and 500 kb were evaluated for potential functional relevance to neurodevelopment and synapse biology.

### 4.5. iPSC Maintenance and Neuronal Differentiation

Geltrex‐coated plates and Essential u (E8) medium (Gibco) with 0.1% penicillin/streptomycin (Fisher Scientific) were used for iPSC maintenance. Cultures were tested weekly for mycoplasma contamination using PCR. All iPSC lines were transfected with the PiggyBac TRE3G‐NGN2‐CMV‐rtTA3G‐Puro construct using Lipofectamine (Fisher Scientific). After transfection, puromycin was added weekly for selection. NGN2 induction allowed for rapid and synchronous neuronal differentiation.

Neuronal differentiation was initiated by plating iPSCs on Geltrex‐coated plates in DMEM/F12 (Gibco, 10565018) supplemented with N2 (Stemcell Technologies), GlutaMAX (200 mM, Life Tech), 0.1% Pen/Strep, 1.5% D‐glucose (20%, Gibco), and 5‐*μ*M ROCK inhibitor (Y‐27632, Tebubio). Differentiation was induced with doxycycline (2 *μ*g/mL, Sigma‐Aldrich), together with SMAD inhibitors LDN‐193189 (100 nM, Stemgent), SB431542 (10 *μ*M, Tocris), and WNT inhibitor XAV939 (2 *μ*M, Stemgent). On DIV2, medium was refreshed, without ROCK inhibitor and supplemented with puromycin (2 *μ*g/mL, Merck‐Millipore). On DIV3, the same conditions were used with the addition of FUDR (10 *μ*M, Sigma‐Aldrich) to suppress proliferating cells. Neurons were replated at DIV4 with Neurobasal Medium (Gibco) with GlutaMAX, 20% D‐glucose, NEAA (Gibco), B27 supplement (Gibco), 0.1% Pen/Strep, 0.5% FBS, and growth factors BDNF, CNTF, and GDNF (10 ng/mL each, Stemcell Technologies).

For RNA analysis, cells were plated on 6‐well plates coated with poly‐L‐ornithine (PLO) and laminin at 300‐k cells per well (two wells per cell line and condition). For patient‐derived cell lines, higher seeding densities were used to compensate for reduced plating efficiency. For protein analysis, 96‐well plates coated with 0.1 mg/mL poly‐D‐lysine and 0.2 mg/mL rat tail collagen (BD Biosciences) were used containing a layer of rat astrocytes. After 4 days, 2‐*μ*M AraC (Sigma‐Aldrich) was added to the astrocytes to halt cell division. This medium was removed before plating 20‐k neurons per well. For the VUS line, twelve 96‐wells were plated per batch and for all other lines six 96‐wells. Twice a week, half of the media was replaced. At DIV9, plates for protein analysis were transduced with phSyn(pr)JRGECO. Half of the VUS wells additionally received pDEST‐hSyn (pr)‐h*STXBP1*(EISS)IRES2‐NLSEGFP to express MUNC18‐1. For NMD inhibition experiments, DIV25 neurons in 6‐well plates were treated with either CHX (100 *μ*M, Sigma‐Aldrich) or the hSMG‐1 inhibitor 11e (1 *μ*M, MedChemExpress, #HY‐124760). The control received 0.14% DMSO. After incubation for 6 h, cells were harvested for RNA analysis.

### 4.6. Calcium Phosphate Transfection

Wild‐type and mutant *STXBP1* genomic fragments were amplified and cloned into the pDESTsplice vector [19] (Addgene #32484) to generate minigene constructs. The WT and VUS constructs were made using the QuickChange method (Stratagene) and DNA was sequence verified. Minigene constructs were transfected into neurons at DIV 15 using calcium phosphate precipitation. Transfection medium was prewarmed in a 37°C incubator, after which 600 *μ*L was added to each well, along with 150 *μ*L of a 5× kynurenic acid solution. For each well, 3‐*μ*g total DNA (minigene construct + mCherry reporter) was mixed with 2.5‐M CaCl_2_, added to 2× HBS buffer and allowed to precipitate for 20 min. The precipitate was then added dropwise to the neurons and incubated for 1 h. After incubation, transfection media was replaced with the original conditioned medium plus 1‐mL fresh medium. Neurons were harvested on DIV18 for RNA analysis.

### 4.7. RNA Analysis

RNA was extracted using the Isolate II RNA Mini Kit (GC Biotech, #BIO‐52073). Before harvest, wells were washed with PBS once. RNA concentrations were measured with Nanodrop, after which 250‐ng RNA was used for cDNA synthesis using the sensiFAST cDNA Synthesis kit (GC Biotech, #BIO‐65054). PCR was performed with Thermo Scientific Phire Hot Start II DNA‐polymerase (Thermo Scientific, #F‐122S) and included 34 cycles of denaturation at 98°C for 10 s, annealing at 60°C for 20 s, and extension at 72°C for 15 s. Primers are shown in Table 1. PCR products were run on 1.5% agarose gel, and DNA bands were collected, purified, and sent for sequencing (Eurofins). qPCR was performed with the QuantStudio 5 Real‐Time PCR System and SensiFAST SYBR Lox‐ROX (GC Biotech, #BIO‐94020) with *GAPDH*, *EIF4G2*, and *UBC* as reference genes.

**Table 1:** Primers used in (q)PCR experiments.

Target	Forward (5 ^′^‐3 ^′^)	Reverse (5 ^′^‐3 ^′^)
*STXBP1* (qPCR)	AGGTGTCCCAGGAAGTC	CTGAGCTCTTTCTGGTACTG
*HNRNPL* (qPCR)	CCCCAATCTCAGTGGACAAG	GCTGCCAGGGTCACCATTG
*GAPDH* (qPCR)	GTTCGACAGTCAGCCGCATC	TCCGTTGACTCCGACCTTCA
*EIF4G2* (qPCR)	AGGACCGCATGTTGGAGATT	TGAGGGGATGGATCCAACTTT
*UBC* (qPCR)	ACGGGACTTGGGTGACTCTA	ATCGCCGAGAAGGGACTACT
*STXBP1* (PCR)	CCGACTGCTAAATACCGGGCTGCACAC	TAGGCATCGAGCTTGTCCTGGATTAGC
Minigene construct (PCR)	CCTGCTCATCCTCTGGGAGC	AGGTCTGAAGGTCACGGGCC

### 4.8. Immunocytochemistry

Neurons were fixed in 3.7% paraformaldehyde (PFA) (Electron Microscopy Sciences) in PBS for 20 min. The cells were then permeabilized using 0.5% Triton X‐100 (Thermo Fisher, #T/3751/08) PBS for 5 min and blocked with 0.1% Triton X‐100 and 2% normal goat serum (NGS) (Thermo Fisher, #11540526) in PBS incubating for 1 h, all at room temperature. Primary antibodies for MUNC18‐1 (rabbit, 1:500, Sigma, #HPA023483), MAP2 (chicken, 1:500, Abcam, #ab5392), synaptophysin‐1 (guinea pig, 1:500, Synaptic Systems, #101308) were added to the wells in blocking buffer. Plates were sealed with parafilm and kept at 4°C overnight, after which the wells were washed three times with PBS. Secondary antibodies Alexa Fluor 647 (goat anti‐rabbit, 1:1000, Fisher Scientific, #A‐21244), Alexa Fluor 546 (goat anti‐chicken, 1:1000, Fisher Scientific, #A‐11040), Alexa Fluor 488 (goat anti‐guinea pig, 1:1000, Fisher Scientific, #A‐11073), and DAPI (1:1000, Carl Roth, #28718‐90‐3) were mixed in blocking buffer and added for incubation for 1 h at room temperature in the dark. After that, wells were washed again 3 times with PBS and stored at 4° until imaging. The NIKON Ti‐Eclipse microscope equipped with a 40× air objective was used to create 16 widefield images per well. Image analysis was performed with an in‐house optimized pipeline using CellProfiler 4 [20]. High‐resolution images of single neurons were acquired by confocal microscopy using the same 40× air objective.

### 4.9. Statistical Analysis Functional Data

A linear mixed‐effects model was fitted to the datasets from the qPCR and fixed imaging assays using biological replicates as a random effect nested within each cell line. The model was used to perform an ANOVA to test for overall differences between conditions. Model assumptions were evaluated by inspecting the residuals. Normality of residuals was assessed using a quantile–quantile (QQ) plot, in which residuals were plotted against a theoretical normal distribution in which the linearity of the points was used to confirm normality. Homoscedasticity and model fit were evaluated with a residuals versus fitted values plot, where residuals were plotted against predicted values and a smoothed trend line (LOESS) was added. A random scatter of points around zero without systematic patterns indicated that the assumptions of constant variance and appropriate model fit were satisfied. Estimated marginal means (EMMs) were calculated for each experimental condition. Planned contrasts were performed to compare specific groups. *p* values were adjusted for multiple comparisons using the Holm method.

### 4.10. EEG Cohort Demographics and Ethics

STXBP1‐RD patients were recruited as part of a multicenter prospective observational study to develop personalized excitation–inhibition targeting treatments for genetic neurodevelopmental disorders [21]. EEG data were collected from patients with STXBP1‐RD, including the index case (*n* = 11 [8 males, 3 females]; age range: 5–12.8 years; mean age [± S.D.] = 7.9 ± 2.2 years), and a group of children with typical development (TDC; *n* = 167; age range: 4–18.9 years; mean age [±S.D.] = 10.8 ± 3.8 years; 59 males, 37 females). Age is reported at the time of EEG recording. TDC were recruited in a dedicated study for controls in neuroscientific studies [15, 22, 23]. The exclusion criteria included a history of behavioral or learning problems, a diagnosis of any neurodevelopmental condition, or any other health issue. Written informed consent was provided by all patients aged > 11 years and legal guardians of children aged < 16 years. The study was approved by the Ethics Review Committee and Institutional Review Boards at the Amsterdam University Medical Centre and Radboud University Medical Center, conducted in accordance with the provisions of the declaration of Helsinki and good clinical practice. The TDC study obtained approval from the Medical Ethical Committee Amsterdam University Medical Center (location AMC, Reference Number NL76915.018.21).

### 4.11. EEG Data Acquisition

EEG recordings used a high‐density 129‐channel system from HydroCel Geodesic with the NetAmps400 amplifier (Magstim‐Electrical Geodesics Inc.). EEG recordings were acquired during awake, eyes‐open rest (EOR) with a duration between 230 and 600 s for patients. The EOR condition was selected because patients frequently have difficulty maintaining their eyes closed for longer durations. The EOR condition maximizes usable recording time and data reliability. For TDC, EEG was acquired with a duration of 300 s, after verbal consent through either a visit to the Emma Children′s Hospital or to our mobile laboratory (“Emma Brain Bus”). The acquisition sampling rate was 1000 Hz with reference electrode Cz and a ground electrode “COM” between CPz and Pz. Electrode impedances were kept below 100 k*Ω*.

### 4.12. EEG Preprocessing

Raw continuous EEG recordings were preprocessed using EEGLAB (v2022.0v, MATLAB R2021b). EEG signals were downsampled to 250 Hz. A 50‐Hz notch filter and a 1–45‐Hz band‐pass filter were applied [24]. Electrodes with poor signal‐to‐noise ratio and flat signals were automatically detected using the Random Sample Consensus algorithm [25], and transient and large‐amplitude artifacts were simultaneously flagged using the Artifact Subspace Reconstruction algorithm [26]. All recordings were manually inspected to decide which flagged channels and artifact‐contaminated segments to reject or retain based on visual assessment. Signals from rejected noisy electrodes were then interpolated using the spherical spline method. Signals were re‐referenced to the average of all electrodes using full‐rank average referencing to preserve data rank and accommodate the interpolated channels. Using independent component analysis (Infomax) algorithm and ICLabel, all recordings were screened for eye movements/blinks, heartbeat and muscle‐related artifacts and projected out from the signals [27, 28]. A final manual inspection was performed to identify and reject any residual artifacts not captured by automated component classification.

### 4.13. EEG Source Reconstruction

Scalp‐level EEG data were projected into cortical source space using L2 minimum norm estimation using MNE‐Python [29, 30]. Biomarkers were computed across 100 cortical patches derived from the Schaefer atlas and subsequently averaged within patches corresponding to the Yeo‐7 atlas of resting‐state networks (default mode, Somato‐motor, salience ventral attention, dorsal attention, visual, limbic, and control networks; for details, see previous studies [15, 31].

### 4.14. EEG Biomarker Estimation and Aggregation

Eleven frequency bands were defined within 1–45 Hz, one band from 1 to 4 Hz and 10 logarithmically spaced bands between 4 and 45 Hz, following the procedure described previously [32]. The resulting frequency bands were 1–4 Hz, 4–5.1 Hz, 5.1–6.5 Hz, 6.5–8.3 Hz, 8.3–10.5 Hz, 10.5–13.4 Hz, 13.4–17 Hz, 17–21.7 Hz, 21.7–27.6 Hz, 27.6–35.2 Hz, and 35.2–44.8 Hz.

The power spectral density (PSD) was computed by filtering the individual Schaefer patch time‐series in the 11 log‐spaced frequency bands from 1 to 45 Hz^32^. For each frequency band, PSD was estimated using Welch′s method with a Hamming window, implemented in MNE software. The length of the fast Fourier transform (FFT) was set to 10 times the sampling frequency, resulting in a frequency resolution of 0.1 Hz. Each segment used a Hamming window equal in length to the FFT length, and window overlap was set to 50% of the FFT length, rounded down to the nearest integer. Relative power was computed as the ratio of each band′s integrated PSD to the total integrated PSD across the full 1–45‐Hz frequency range and expressed in percentage (%).

DFA quantifies long‐range temporal dependencies in neural oscillations, which reflect excitation–inhibition dynamics at the network level [32, 33]. Autocorrelation in the amplitude envelope of each frequency band was quantified by the DFA exponent, which relates the mean fluctuation in the amplitude to increasing time‐window sizes within a chosen time‐scale range [33, 34]. A DFA exponent of 0.5 indicates an uncorrelated random signal, whereas values > 0.5 denote positive autocorrelation whose strength grows with the exponent. Before computing DFA, each signal was bandpass filtered into 11 frequency bands, and the amplitude envelope for each band was extracted using a Hilbert transform. For each frequency band, the DFA exponent was obtained as the slope of the line relating the log‐fluctuation to the log‐window size, fitted using the time‐scale ranges.

The network‐level balance between excitation and inhibition was quantified using fE/I, which captures the covariance between the signal′s amplitude and its autocorrelation [35]. The fE/I value < 1 indicates inhibitory dominance in the signal, > 1 excitatory dominance, and 1 a balanced E/I ratio. Before computing fE/I, each signal was bandpass filtered into 11 frequency bands, and the amplitude envelope for each band was extracted using a Hilbert transform (also via the MNE software). For each band, the temporal variation in amplitude autocorrelation was approximated by windowed amplitude fluctuations, and fE/I was calculated as 1 minus the Pearson correlation between the windowed amplitudes and their fluctuations. The DFA exponent was computed as described above, and fE/I was set to missing (NaN) whenever DFA < 0.6, because the covariance estimate is only meaningful for signals with substantial autocorrelation [32, 35]. A step‐by‐step description of this algorithm is described previously [32].

Relative power, DFA exponents, and fE/I values were computed across 11 logarithmically spaced frequency bins and grouped into five canonical bands: delta (*δ*: 1–4 Hz), theta (*θ*: 4–8.2 Hz), alpha (*α*: 8.2–13.3 Hz), beta (*β*: 13.3–21.7 Hz), and high beta/gamma (*β*‐*γ*: 21.7–44.8 Hz). For each of the 100 Schaefer cortical parcels, relative power was sum aggregated, whereas DFA exponents and fE/I were median aggregated within each canonical band. The network‐level estimates for each EEG biomarker were computed as the median across parcels associated with each Yeo network, separately for the left and right hemispheres [31]. Global network‐level estimates were derived by computing an average across hemispheres. This resulted in a final feature matrix of 35 EEG features per biomarker per subject, across five canonical bands and seven Yeo networks. The 35 features provided a compact yet comprehensive representation of spectro‐spatial qEEG organization while ensuring the feature count remained below the TDC sample size (*n* = 167), a prerequisite for stable covariance estimation and principal component analysis (PCA) [15].

### 4.15. Normative Low Dimensional EEG Feature Space

We constructed a low‐dimensional normative EEG feature space from TDC cohort using sparse PCA [15]. Characterizing typical patterns of EEG organization during healthy development is essential for identifying meaningful deviations in patient populations. The reference space captured the spectral and spatial distribution of relative power, DFA exponents, and fE/I across canonical cortical networks and frequency bands. Median and interquartile range scaling parameters were estimated on the TDC and applied to both TDC and STXBP1‐RD datasets. A sparsity constraint was imposed on the loading vectors which forces many feature loadings to zero, yielding components dominated by a subset of features rather than dense linear combinations of all 35 features [36, 37].

### 4.16. Quantification of TDC and Patient Mahalanobis Distances

For each biomarker, pairs of principal components were examined in two‐dimensional normative spaces. The Minimum Covariance Determinant estimator was used to compute robust centers and covariance matrices [38]. A global center based on the entire TDC cohort (*n* = 167) was estimated. Individual STXBP1‐RD patient profiles were projected into the normative space and their deviation quantified using Mahalanobis distances [15, 39, 40]. MD measures how far a subject′s multivariate qEEG point lies from the normative center after taking the normative covariance structure into account. A higher MD indicates a profile that is statistically more distant from the normative center, reflecting greater neurophysiological abnormality [15, 40].

## Author Contributions

S.K. performed all functional cellular experiments, including cell culture, transfections, and RNA and protein analyses. S.A. and J.R.R. acquired patient EEG data, and A.S. conducted the qEEG analyses. J.J.S. performed clinical assessments, including IQ and adaptive behavior. H.B. designed the N = You multimodal assessment pipeline and supervised clinical assessments, K.L‐H. supervised qEEG analyses, R.F.T. supervised cellular analyses, and M.V. supervised the study; M.M‐I. supervised the study and maintained patient family communication. S.K. and M.V. wrote the manuscript with input from all authors.

## Funding

This study was supported by the ZonMw (10.13039/501100001826) (10250022110003, 733051137) and Nederlandse Organisatie voor Wetenschappelijk Onderzoek (10.13039/501100003246) (024.004.012).

## Disclosure

All authors approved the final version.

## Conflicts of Interest

The authors declare no conflicts of interest. M.V. and R.F.T. established Neurospector, an academic CRO that offers commercial protein and mRNA analyses for STXBP1, similar to the analyses reported here and receive personal compensation for such work.

## Data Availability

Data are available from the corresponding authors on reasonable request.
